# Category Exemplar Production Norms for Hong Kong Cantonese: Instance Probabilities and Word Familiarity

**DOI:** 10.3389/fpsyg.2021.657706

**Published:** 2021-08-09

**Authors:** Bing Li, Qiduo Lin, Hoi Yan Mak, Ovid J. L. Tzeng, Chih-Mao Huang, Hsu-Wen Huang

**Affiliations:** ^1^Department of Linguistics and Translation, City University of Hong Kong, Kowloon, Hong Kong; ^2^Hong Kong Institute for Advanced Study, City University of Hong Kong, Kowloon, Hong Kong; ^3^Department of Biological Science and Technology, National Yang Ming Chiao Tung University, Hsinchu, Taiwan; ^4^Department of Educational Psychology and Counseling, National Taiwan Normal University, Taipei, Taiwan

**Keywords:** Hong Kong Cantonese, norm, semantic category, typicality, familiarity, lexicon

## Abstract

The lexical system of Hong Kong Cantonese has been heavily shaped by the local trilingual environment. The development of cultural- and language-specific norms for Hong Kong Cantonese is fundamental for understanding how the speaker population organize semantic memory, how they utilize their semantic resources, and what information processing strategies they use for the retrieval of semantic knowledge. This study presents a normative database of 72 lexical categories in Hong Kong Cantonese produced by native speakers in a category exemplar production task. Exemplars are enlisted under a category label, along with the instance probabilities and word familiarity scores. Possible English equivalents are given to the exemplars for the convenience of non-HKC speaker researchers. Statistics on categories were further extracted to capture the heterogeneity of the categories: the total number of valid exemplars, the number of exemplars covering 90% of the occurrence and the probabilities of the most frequent exemplars in each category. The database offers a direct lexical sketch of the vocabulary of modern Hong Kong Cantonese in a categorical structure. The category-exemplar lists and the comparative statistics together lay the foundations for further investigations on the Hong Kong Cantonese speaking population from multiple disciplines, such as the structure of semantic knowledge, the time-course of knowledge access, and the processing strategies of young adults. Results of this norm can be also used as a benchmark for other age groups. The database can serve as a crucial resource for establishing initial screening tests to assess the cognitive and psychological functioning of the Cantonese-speaking Hong Kong population in both educational and clinical settings. In sum, this normative study provides a fundamental resource for future studies on language processing mechanisms of Hong Kong Cantonese speaking population, as well as language studies and other cross-language/culture studies on Hong Kong Cantonese.

## Introduction

Being categorical is a fundamental property of our knowledge of the world (Barsalou, 2003). Categorization, i.e., sorting things based on their shared components, is an important information processing activity embedded in our perceptions of the surroundings and interactions with them. Research has shown that the categorical frame of semantic knowledge and the uneven statuses of category members profoundly influence our language and information processing (Rips et al., 1973; Rosch and Mervis, 1975; Mervis and Rosch, 1981), to a degree that being categorical pervades the way we think and live in the social context (Marx and Ko, 2012). To understand the categorical structure of knowledge, both psychologists and linguists have pursued inquiries such as *what is (or is not) a type of X, and why?* and *is item X considered a good category member, and why?* (Lakoff, 1973; Smith et al., 1974; Rosch, 1975).

There is a graded structure within categories (Lakoff, 1973; Rosch, 1975; Barsalou, 1985), which consists of a core that includes the most representative (i.e., high-typical) examples surrounded by the exemplars which are less representative (i.e., low-typical). In other words, category members are not equal in terms of the “goodness” of their membership. This non-equivalence of category members (Mervis and Rosch, 1981) is reflected in the probability that a member will be recalled in production tasks, or in the subjective rating of a proposed category member’s degree of typicality (Rosch, 1975). Nevertheless, the frequency of which an exemplar is mentioned in a production task is significantly correlated with its typicality rating (Mervis et al., 1976; Mervis and Rosch, 1981). Therefore, the frequency results from a category exemplar production task are also reliable for indexing exemplar typicality. In this way, the exemplar production task conveniently provides both the exemplars and their typicality measurements at the same time.

Higher typicality is usually associated with higher processing efficiency in terms of production probability, accuracy, and reaction time (for a review, see Rosch et al., 1976, and many others). Processing efficiency (i.e., the typicality effect) has been observed and verified in multiple categorization tasks in a variety of studies, such as category acquisition, exemplar production, and membership verification (also reviewed in Barsalou, 1985). For example, in membership verification tasks, when a subject is asked to verify a statement in a sentence such as “X is (not) a (kind of) Y” as rapidly as possible, more typical or representative items are processed with shorter reaction times regardless of the statement’s veracity (Mervis and Rosch, 1981). Only typical category-instance pairs are facilitated by the category name in the same-or-different matching task (reviewed in Rosch et al., 1976). Developmentally, children integrate typical instances of categories into their language and conceptual systems before atypical instances (Bjorklund et al., 1983). However, it has also been suggested that processing efficiency may be attributable to the high familiarity of a word (often measured by word frequency) rather than its high typicality, because highly familiar exemplars are generally more salient in all daily language use scenarios. The more familiarized exemplars are recalled more often and rated as more typical (Janczura and Nelson, 1999); the fact that the exemplars rated as less typical may be due to the low familiarity of a target word (Malt and Smith, 1982). Furthermore, it has been suggested that cross-cultural discrepancy of typicality may be due to the general cultural familiarity (Schwanenflugel and Rey, 1986). The literature on the underlying mechanism of processing efficiency and the possible interaction of typicality and familiarity presents contradictory evidence with no clear conclusions (Rosch et al., 1976; McCloskey, 1980; also see Murphy, 2002, for a review).

The typicality effect interacts with other categorical properties of exemplars, such as the categories they belong to. To further identify and observe typicality effect in interaction, categories can be further split into subgroups of different types (thus adding an extra stratum to categories), and the effects of the contrasting characteristics of the category subgroups can be investigated. For example, in studies of language deficits that aimed to identify the selective impairment of domain-specific knowledge attributable to brain damage, differences have been observed in language processing with respect to inanimate vs. animate words (Caramazza and Shelton, 1998), concrete vs. abstract words (Catricalà et al., 2014), and words from well-defined/closed vs. fuzzy-boundary categories (Kiran and Johnson, 2008). The outcomes of these studies highlight the necessity for and research potential of a normative database with comprehensive coverage of categories and exemplars and, ideally, with reliable prescriptive statistics.

Language materials in the target language are fundamental to research and experiments such as those described above. These materials are usually presented in a database containing a considerable number of categories with exemplars produced by native speakers responding to a category cue. Over time, the number of categories included keeps expanding to meet the demand for a larger and more heterogeneous coverage, as demonstrated by the expansion of databases over time; for example, the original “Connecticut norms” included 43 categories (Cohen et al., 1957), which were expanded to 56 categories by Battig and Montague (1969) and then to 106 categories by McEvoy and Nelson (1982). Norms have also been replicated and constantly updated to capture the conceptual shifts and drifts over time and socio-cultural differences (Van Overschelde et al., 2004), and also in languages besides English (e.g., Bueno and Megherbi, 2009 in French; Storms, 2001 in Flemish). The exemplars in each category and their comparative statistics provide a detailed and rich image of the semantic resource in a given target language. These categorical data are usually collected from native speakers who are healthy young adults (e.g., college students). The results are then used as benchmarks for comparison with other age groups (i.e., children and older adults) or adults with impaired cognitive functions and language abilities. For example, a series of studies demonstrated that the use of atypical exemplars from various categories is an effective training method for patients with aphasia (Kiran et al., 2011).

Given the time and expense associated with a norming study, it is not surprising that few appropriate non-English databases are available. The current study aims to address this issue for Hong Kong Cantonese, which is the lingua franca among Hong Kong Chinese population. The spoken and written forms of Hong Kong Cantonese have emerged from the combination of Hong Kong’s special socioeconomic status, its colonial history and the inevitable language contact with Mandarin Chinese (*Putonghua*) since the handover from Great Britain in 1997. Hong Kong Cantonese is distinct from other varieties of Chinese with similar or even mutually understood pronunciations (e.g., Cantonese spoken in Guangdong) and consistent writing systems (e.g., Taiwan Mandarin, which is also written in Traditional Chinese characters). At the lexical level, Hong Kong Cantonese has been strongly shaped by a trilingual environment in which Cantonese, English, and Putonghua are used simultaneously (sometimes even within the composition of a word). Specifically, language elements from English of various lengths and units were fused into the daily usage both in non-formal writings and in speech (i.e., Cantonese-English code switching, Li and Lee, 2004), with phonetic borrowing and transliteration used as tools and resources (Li, 2000). For example, the transliteration of strawberry in Hong Kong Cantonese results in “士多啤梨” (“*si6 do1 be1 lei4*,” “strawberry”). This representation is understood by most Cantonese speakers in the adjacent province, and even some Mandarin speakers. Nonetheless, the formal and preferred name of the fruit for Cantonese speakers outside Hong Kong is “草莓,” “*cǎo méi*” which is often used as the written form of “strawberry” in Hong Kong Cantonese (but rarely as the colloquial form). In addition, certain concepts, and hence the words and phrases representing them, only exist in Hong Kong Cantonese. For example, the concept and term “公屋” (“*gung1 uk1*” “public/government-owned housing”) is used by Hong Kong Cantonese but not by Guangzhou Cantonese speakers. The word is not in the lexical inventory of Guangzhou Cantonese speakers, but they would not find it difficult to read the Chinese characters and pronounce them in Cantonese, and they could probably guess the meaning.

In light of these considerations, this study conducted two experiments to establish a categorical normative database of Hong Kong Cantonese consisting of multiple categories and exemplars. One is a category exemplar production task, and the other one is the familiarity rating task. Within each category, the instance probability of every exemplar and its familiarity rating score was calculated. Furthermore, various indices associated with the recalled exemplars in each category were complied to capture the heterogeneity across the categories.

## Materials and Methods

### Experiment 1: Category Exemplar Production Task

#### Materials

This experiment included 84 categories. The full list of categories was adapted and modified from one of the author’s unpublished work and a cross-language sociolinguistic norm study (Yoon et al., 2004). All lexical forms of the category names and the written materials were advised and verified by two native Hong Kong Cantonese speakers. A pilot study has been conducted to ensure that categories are “productive.”

#### Participants

Forty young adults aged between 18 and 24 years (mean 20.2 years; 20 females) participated in this study. All participants were native Hong Kong Cantonese speakers who were raised in Hong Kong up to the age of 18, with Cantonese reported as their mother tongue. The participants completed a language ability questionnaire in which they were instructed to self-evaluate their Cantonese reading, listening, speaking, and writing proficiency levels. Their Cantonese language abilities of all the above four aspects were reported as proficient. However, all of them would have been exposed to a mixed rather than a monolingual language background because of the “bi-literacy and tri-lingualism” language education policy imposed by the Education Bureau of Hong Kong. All of the participants had a normal reading ability and no reported cognitive impairments. The study was approved by the Institutional Review Board of the City University of Hong Kong, and all of the participants provided written informed consent prior to their participation.

#### Procedures

Eighty four categories (i.e., trials) were included in the experiment. The trial order was randomized. Each participant completed 84 trials which divided into two blocks of 42 trials each, considering the time consumption and fatigue of completion. The participants were asked to produce three exemplars for each category at their own pace. They were instructed to produce the three most representative examples they could think of for a particular category, following the order of the “goodness” of category membership (best fit, second-best fit, third-best fit). The responses were preferably words comprising two or three Chinese characters. The participants input their responses into the interactive online survey form using the provided desktop computers in a controlled and supervised environment. An interactive page for each category began with the cue: “A type of AAA” (where AAA represents the category name). The participants were then prompted by the text following the text line of the category name: “*1. The best fit that comes to mind*,” “*2. The second-best fit that comes to mind*,” and “*3. The third-best fit that comes to mind.*” They were asked to fill in all three slots, with no skipping. Each participant took a short break between the two trial blocks.

### Compilation of the Exemplars From the Individual Responses for Experiment 2

Data cleaning and item combining were manually applied to individual cases. Typos were identified and corrected; for example, “債卷” (typo) was changed to “債券”(corrected, “*zaai3 hyun3*,” “bond,” as a response to “a kind of investment tool”). The mixed usage of simplified Chinese characters was adjusted; for instance, “生气” (Chinese-Simplified) was changed to “生氣” (Chinese-Traditional, “*sang1 hei3*,” “angry,” in response to “a mood state”). Allographs were unified; for example, “雞” and “鷄” (allographs for “chicken,” “*gai1*”) were merged to yield “雞.” Variations of words used to describe a very similar or identical concept were merged into a common form and treated as identical, as in the case for “乳牛”(“*jyu5 ngau4*”, milk cow) and “奶牛” (“*naai5 ngau4*”, “milk cow”), which were deemed to describe the identical concept of “milk cow” as a response to “a kind of farm animal.” These variations are due to differences between formal and informal speech rather than to conceptual differences (there is no analogous example available in English).

### Experiment 2: Familiarity Rating

Understanding whether the familiarity of a word impacts its categorical typicality is an essential step toward understanding how semantic knowledge is organized. This experiment assessed the familiarity of each concept in the general context of participants’ daily lives. Participants were instructed to rate how often they encountered a target word (an exemplar from Experiment 1) in all the life scenarios, instead of being under a specific category. Note that here the categorical information was not given to the word to be rated.

#### Materials

The participants provided familiarity ratings for the words generated in the first experiment. Categorical information from the previous task was given only if the exemplar was potentially ambiguous, by referring two different concepts belonging to two categories. For example, “杜鵑” (“*dou6 gyun1*”) can refer either to the rhododendron flower (“杜鵑花,” “*dou6 gyun1 faa1*,” rhododendron) or a cuckoo bird (“杜鵑鳥,” “*dou6 gyun1 niu5*,” cuckoo, a very common image in traditional poetic rhetoric). In such cases, categorical information (often indicated by a single Chinese character, such as “花” for “flower” and “鳥” for “bird”) was given in parentheses at the end of the target word for disambiguation. For example, the item was presented as “杜鵑（花）” [“*dou6 gyun1(faa1)*,” rhododendron] if the target word was from the category “a kind of flower.”

#### Participants

Forty additional young adults aged 18–23 years (mean 20.3 years; females = 20) were recruited for this study. None of these participants had prior exposure to the test materials. The recruitment process and eligibility criteria for the participants were the same as those in Experiment 1. All of the participants were native Hong Kong Cantonese speakers with normal reading ability and no reported psychiatric disorders. The study was approved by the Institutional Review Board of the City University of Hong Kong, and the participants provided written informed consent prior to their participation.

#### Procedures

The whole set of target words was randomized and split into two lists. Each participant provided familiarity ratings for one list of approximately 650 words. The test environment was monitored as described for Experiment 1.

The participants were asked to rate the familiarity of the exemplars using a 7-point scale ranging from 1, “extremely unfamiliar,” to 7, “very familiar.” The participants were instructed to rate the target words based on their subjective daily personal experiences.

## Results

### Measurements: Categories, Exemplars, and Familiarity

The database included 1298 items in 72 categories. The results from the two experiments were integrated and presented in tables, one for each category. A representative example is shown in Table 1. A word code (*Word Code*) was assigned to every exemplar under a specific category using the format HKC (the acronym for Hong Kong Category) followed by a 3-digit category code (e.g., “001” for “a kind of farm animal”) and a 2-digit exemplar code. The exemplar (*Word*) was numbered to indicate the descending rank of total probabilities within the category. “*Slot 1*,” “*Slot 2*,” and “*Slot 3*” indicate the probabilities of a given exemplar being allocated on the best/second-best/third-best slot. The probability on a slot is the number of mentions in the given slot divided by the total number of eligible entries for that slot, rounded to three decimal places. “*Total*” is the instance probability of an exemplar, regardless of the slot. “*Accumulative*” is the summed instance probability of the exemplar and that of all its precedents. Logically, this value increases with each exemplar in the category until it reaches 1.000 at the last exemplar. “*Familiarity*” is the average of all rating scores given by all participants who viewed that exemplar. *Possible English Equivalents* are listed as the possible corresponding concepts in the English, for the convenience of the researchers who are interested in further studies on cross-language comparisons.

**TABLE 1:** Category of “a farm animal” (HKC001).

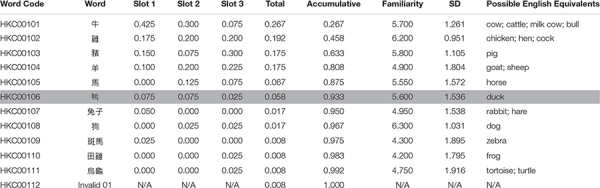

*The exemplar in a category at which the Accumulative reaches 0.90 is shadded. SD is the standard deviation of the Familiarity score.*

At the end of each table, *Invalid xx* is a designed virtual exemplar indicating sum of the probabilities of all invalid responses, where “xx” is the category code. Invalid responses may be due to mistyping, misunderstanding of the category name, or lack of knowledge of the category (as mentioned in the data compiling and cleaning section). In some cases, when the participants were unable to think of a word, they repeated a response or rephrased it to an interchangeable lexical item. For example, both “石屎” (“*sek6 si2*”) and “英泥” (“*jing1 nai4*”) refer to “cement” in the category of “construction materials”; only that “石屎” (“*sek6 si2*”) is more colloquial. In other cases, participants generated non-referring items, such as “square” or “round” in the category of “natural geographical feature,” indicating unfamiliarity of geographical terminology; while participants in other studies were able to produce more relevant and referring terms such as “mountain” and “lake,” as the cases in other norm studies (Van Overschelde et al., 2004). Such non-referring items were considered as invalid responses. The probabilities of the invalid responses for the individual slots were not considered informative and thus were omitted from the table by designating the values as “N/A.” No familiarity scores were associated with the invalid responses, and hence this field was also marked as “N/A.” When there was no invalid response in a given category, the *Total* of the *Invalid xx* was 0.000, and when the *Total* of *Invalid xx* reached 0.500, the category would be excluded from the final table. Invalid responses may have occurred because the category and its related information were unfamiliar or unavailable to Hong Kong Cantonese speakers; therefore, categories with more than 50% invalid responses were discarded because they were not able to represent the consensus of semantic knowledge in the population. Twelve categories (e.g., a kind of natural geographical feature) were discarded from the final list (see the Appendix). A total of 72 categories were included in the following analyses.

### Reliability of the Measurements

#### Split-Half Correlations

To ensure the consistency and reliability of the data, split-half correlations were applied and corrected using the Spearman–Brown formula on both *Slot1* and *Total* with data from the 40 participants split into the first half and second half. For *Slot1*, the split-half correlation was generally very high (median = 0.911), although three categories were lower than the threshold of 0.700: “Toy” (*r* = 0.490), “Fuel” (*r* = 0.676), and “NGO” (*r* = 0.596). For *Total*, the split-half correlation was very high for each category (median = 0.945, range = [0.840 −0.993]). For the familiarity results, an identical split-half correlation was applied and corrected using the Spearman–Brown formula, and the rating results from the two subgroups were highly correlated (*r* = 0.915). The high correlations show that the data of the two experiments were reliable and consistent.

### Slot 1 and Total

Previous studies have suggested that the most frequently generated exemplar within a category is the most typical and hence the central member of that category (Barsalou, 1985). The more central an exemplar, the faster and more frequently it is recalled as a category exemplar, as the search process follows a fixed order (Rosch, 1973). Given this logic, the exemplars that are recalled most frequently (higher *Total*) should also be recalled as the first responses (best-fit) more frequently (in *Slot1*). To examine this hypothesis, the Pearson’s correlation coefficient of the two sets of probabilities (*Slot1* and *Total*) of the exemplars was calculated for each category as shown in Table 2. Note that only the exemplars that had been mentioned in Slot 1 at least once (i.e., the value of *Slot1* was >0.000) were included in the correlation analysis. Besides the number of included exemplars in the correlation analysis *n*, the total number of exemplars listed in a category N was also presented. Significant positive correlations were observed for 62 out of 72 categories and marked in the table, confirming that most frequently recalled exemplars are also likely to be mentioned first for the majority of the categories.

**TABLE 2:** *Total-Slot1* correlations on all categories.

Category Code	Pearson’s r	*n* ^1^	Valid Exemplars^2^
HKC001	0.852*	7	11
HKC002	0.801**	10	17
HKC003	0.865**	10	20
HKC004	0.955**	8	19
HKC005	0.830**	11	16
HKC006	0.875**	9	11
HKC007	0.813*	7	16
HKC008	0.927**	8	17
HKC009	0.861*	7	12
HKC010	0.897**	10	15
HKC011	0.892**	9	14
HKC012	0.905**	12	25
HKC013	0.698	5	12
HKC014	0.961**	7	14
HKC015	0.830**	9	18
HKC016	0.958**	6	17
HKC017	0.904**	9	24
HKC018	0.943**	12	20
HKC019	0.965**	13	27
HKC020	0.977**	11	24
HKC021	0.827**	13	16
HKC022	0.931**	7	18
HKC023	0.785*	9	19
HKC024	0.822**	11	20
HKC025	0.912**	14	23
HKC026	0.633	7	16
HKC027	0.936**	7	19
HKC028	0.837**	10	23
HKC029	0.959**	16	32
HKC030	0.847**	13	25
HKC031	0.854**	10	15
HKC032	0.943**	7	14
HKC033	0.918**	11	28
HKC034	0.890**	13	23
HKC035	0.858**	12	22
HKC036	0.946**	10	20
HKC037	0.981**	10	26
HKC038	0.763*	8	17
HKC039	0.770*	8	18
HKC040	0.793*	8	13
HKC041	0.844**	9	13
HKC042	0.897**	11	14
HKC043	0.935**	6	14
HKC044	0.905*	5	19
HKC045	0.821*	6	9
HKC046	0.787	5	14
HKC047	0.674*	10	28
HKC048	0.245	4	9
HKC049	0.893**	7	14
HKC050	0.826**	18	31
HKC051	0.758	3	19
HKC052	0.634	5	12
HKC053	0.667	6	16
HKC054	0.970**	9	25
HKC055	0.980*	4	13
HKC056	0.907**	8	14
HKC057	0.915*	5	18
HKC058	0.625	9	13
HKC059	0.929**	8	12
HKC060	0.879**	10	26
HKC061	0.954**	9	32
HKC062	0.606*	13	15
HKC063	0.802*	7	11
HKC064	0.911**	16	25
HKC065	0.808	5	12
HKC066	0.791	5	15
HKC067	0.830**	11	18
HKC068	0.779**	10	22
HKC069	0.886*	5	10
HKC070	0.740**	13	26
HKC071	0.958*	5	12
HKC072	0.836**	8	10

*^1^Only the exemplars of which *Slot1* > 0.000 are included in the correlation analysis. The *n* is thus the number of exemplars mentioned at least once on *Slot1* in a category, while the ones not recalled on *Slot1* (*Slot1* = 0.000) are excluded in the correlation.*

^2^The actual number of all the valid exemplars listed in a category.

*p < 0.05, **p < 0.01.

### Familiarity and Total

Here, familiarity is defined as the average score of participants’ subjective ratings of the frequency of encountering an exemplar across all daily contexts and scenarios. The participants were not given categorical information about the target words in the familiarity experiment (except in cases of ambiguity), which differs from the procedures in some studies (e.g., Hampton and Gardiner, 1983). Familiarity measures how often the target word (the written form of a concept) is experienced in a general context, among other words which are not necessarily from the same category. In experiment 2, we asked participants to rate how often they experienced (by hearing, reading or using, etc.) the word “狗” (“*gau2*,” dog) in their daily lives, instead of asking them to rate how often they had experienced it as “a kind of domestic pet.” This approach of avoiding the co-occurrence of the exemplar and its category limited the potential interaction between general familiarity with the concept itself, as well as familiarity with the concept cued by a certain category name. To further examine the relationship between the probability the production probability of an exemplar under a given category (*Total*) and the familiarity of the concept in general (*Familiarity*), the correlations between *Total* and *Familiarity* were calculated within each category (see Table 3). Each category contains different numbers of exemplars in this analysis, and for each category there is a correlation *r* and a corresponding *p*-value. No significant correlations were identified for the majority of categories (51 of 72), indicating that more frequently experienced concepts were not necessarily produced more frequently in response to a category cue. As mentioned earlier, instance probability is a legitimate a measurement of exemplar typicality, and the familiarity of a word is highly correlated with its frequency. Thus, the results of the current study are in line with those of previous studies (Mervis et al., 1976; Rosch et al., 1976).

**TABLE 3:** Correlations between total (the probability of being recalled, indexing typicality) and familiarity of exemplars, for each category.

Category Code	Category Name	Pearson’s r	Number of Exemplars in Category (N)	*p*-value
HKC001	農場動物Farm Animal	0.481	11	0.134
HKC002	調味料Spice	0.560*	17	0.019
HKC003	家用電器 Household Appliance	0.461*	20	0.041
HKC004	汽車零件 Car Part	0.302	19	0.223
HKC005	浴室用品 Bath Utensil	0.317	16	0.231
HKC006	寵物 Pet	0.868**	11	< 0.001
HKC007	酒精飲料 Alcohol Drink	0.489	16	0.055
HKC008	罪行 Crime	0.237	17	0.36
HKC009	建築材料 Construction Material	0.129	12	0.689
HKC010	布料 Fabric	0.100	15	0.724
HKC011	化粧品 Makeup Product	0.305	14	0.29
HKC012	鳥類 Bird	0.440*	25	0.028
HKC013	乳製品 Dairy Product	0.350	12	0.265
HKC014	舞蹈 Dance	0.497	14	0.071
HKC015	水果 Fruit	0.539*	18	0.021
HKC016	消防器材 Firefighting Supply	0.385	17	0.126
HKC017	花 Flower	0.359	24	0.085
HKC018	民間藝術 Folk Art	0.329	20	0.156
HKC019	野生動物 Wild Animal	0.311	27	0.114
HKC020	疾病 Disease	0.425*	24	0.043
HKC021	茶葉 Tea	0.484	16	0.058
HKC022	家俬 Furniture	0.044	19	0.863
HKC023	房屋類型 Housing Type	0.375	19	0.114
HKC024	昆蟲 Insect	0.528*	20	0.017
HKC025	廚房用具 Kitchen Utensil	−0.066	23	0.766
HKC026	金屬 Metal	0.565*	17	0.023
HKC027	零食 Snack	0.272	19	0.259
HKC028	樂器 Musical Instrument	0.558**	23	0.006
HKC029	職業 Profession	0.438*	32	0.014
HKC030	人體器官 Human Organ	0.095	25	0.652
HKC031	寶石 Gem	0.562*	15	0.029
HKC032	祭祀用品 Ancestral Worship Item	−0.082	14	0.781
HKC033	運動 Sport	0.387*	28	0.042
HKC034	園藝工具 Gardening Tool	0.248	23	0.254
HKC035	玩具 Toy	0.241	22	0.279
HKC036	蔬菜 Vegetable	0.408	20	0.074
HKC037	武器 Weapon	0.277	26	0.17
HKC038	天文現象 Astronomical Phenomena	−0.096	17	0.714
HKC039	文具 Stationery	0.434	18	0.072
HKC040	沐浴用品 Bath Product	0.514	13	0.073
HKC041	器皿 Container	0.477	13	0.1
HKC042	清潔工具 Cleaning Tool	0.225	14	0.44
HKC043	照明工具 Lighting Appliance	0.264	14	0.363
HKC044	投資工具 Investment Tool	0.443	19	0.057
HKC045	交通工具 Transportation	0.829**	9	0.006
HKC046	形狀 Shape	0.493	14	0.073
HKC047	情緒 Emotional State	0.345	28	0.078
HKC048	餐具 Tableware	0.116	9	0.766
HKC049	急救用品 First Aid Supply	0.343	14	0.229
HKC050	休閒活動 Recreational Activity	0.322	31	0.077
HKC051	街頭小食 Street Food	0.322	19	0.179
HKC052	語言 Language	0.886**	12	< 0.001
HKC053	電影類型 Movie	0.068	16	0.801
HKC054	營養補充劑 Nutritional Supplement	0.304	25	0.139
HKC055	標點符號 Punctuation Mark	0.739**	13	0.004
HKC056	貨幣 Currency	0.644*	14	0.013
HKC057	茶樓點心 Teahouse DimSum	0.427	18	0.077
HKC058	燃料 Fuel d	0.139	13	0.65
HKC059	鞋款 Shoe	0.61*	12	0.035
HKC060	藝術品 Artwork	0.248	26	0.221
HKC061	香港景點 Sightseeing Spot	0.338	32	0.058
HKC062	服飾配件 Fashion Accessory	0.290	15	0.294
HKC063	年齡組別 Age Group	0.204	11	0.548
HKC064	公益團體 NGO	0.515*	25	0.01
HKC065	電子產品 Electronic Device	0.662*	12	0.019
HKC066	度量工具 Measuring Tool	0.348	15	0.203
HKC067	糖水 Sweet Soup/Tong Sui	0.347	17	0.158
HKC068	海洋生物 Marine Animal	0.337	22	0.125
HKC069	時間單位 Time Unit	0.374	10	0.287
HKC070	長輩稱呼 Name for Addressing Elder Relatives	0.510**	26	0.008
HKC071	重量單位 Weight Unit	0.309	12	0.354
HKC072	天然能源 Natural Energy Resource	0.661*	10	0.038

*p < 0.05, **p < 0.01.

The familiarity-based explanation of faster and more accurate processing for typical exemplars could be due to the generally high degree of familiarity of the concepts (Ashcraft, 1978), and familiarity confounds to the pattern of experimental results (e.g., processing efficiency) that argued for a semantic memory model (McCloskey, 1980). However, even if word familiarity is an important determinant of typicality, it cannot account for all of the variance in typicality ratings (Rosch et al., 1976).

In this study, all combinations of “*Familiarity*” and “*Total*” were observed (high-F and low-T; high-F and high-T; low-F and high-T; low-F and low-T) for the exemplars. For example, dog (“狗,” *gau2*) was a highly familiar concept among the participants (6.30 out of 7.00, higher than the category average of 5.30), but was retrieved as a low typical member in the category “a kind of farm animal” (*Slot1* = 0.000, *Slot2* = 0.025, *Slot3* = 0.025, *Total* = 0.017). In contrast, solar eclipse (“日蝕,” *jat6 sik6*) was a far less familiar concept (4.25 of 7.00, lower than the category average of 4.81), but was the top-mentioned exemplar in the category of “an astronomical phenomenon” (*Slot1* = 0.425, *Slot2* = 0.150, *Slot3* = 0.05, *Total* = 0.208).

Previous norming studies have often found familiarity (or word frequency) to be correlated with indices of typicality, such as overall frequency, first-occurrence, and mean rank (Montefinese et al., 2012), because typicality and familiarity are both associated with the ease of production of an exemplar. The non-correlation discrepancy may be due to the experimental designs used in the current study. In Experiment 1, the number of category responses was restricted to three, so all three responses were more likely to be highly familiar items, though their instance probabilities would still differ. In Experiment 2, the familiarity ratings were provided without the category context or other category items, though familiarity ratings were done within a category in some other studies (e.g., Hampton and Gardiner, 1983). The familiarity ratings in Hampton’s work are of more comparative and relative results among category members.

### Properties of Categories

The comparative statistical indices of the categories are shown in Table 4, where “*Valid Exemplars*” represents the number of valid exemplars listed in the category. “*Exemplars to 0.90 Coverage*” is defined as the number of exemplars covering 90% of the occurrences of all valid entries. “*0.90 Coverage%*” is calculated as *Exemplars to 0.90 Coverage* divided by *Valid Exemplars*. “*Invalid Exemplars%*” is the proportion of invalid responses in a category, the same as *Total* of *Invalid xx* in Table 1. “*First Exemplar Total%*” is the *Total* probability of the top-ranked exemplar in that category, which indicates the degree of dominance of that exemplar and how typicality congregates in that category. “*Average Familiarity*” is the average familiarity score of all of the valid exemplars in a category, along with its standard deviation.

**TABLE 4:** Compiled statistics for all categories.

Category Code	Category Name	Valid Exemplars	Exemplars to 0.90 Coverage	0.90 Coverage%	Invalid Exemplars%	First Exemplar Total%	Avg. Familiarity
HKC001	農場動物 Farm Animal	11	6	0.545	0.008	0.267	5.295 (±0.719)
HKC002	調味料 Spice	17	9	0.529	0.000	0.217	5.268 (±0.639)
HKC003	家用電器 Household Appliance	20	11	0.550	0.000	0.225	5.705 (±0.536)
HKC004	汽車零件 Car Part	19	19	1.000	0.117	0.317	4.389 (±0.802)
HKC005	浴室用品 Bath Utensil	16	13	0.813	0.058	0.233	5.538 (±0.608)
HKC006	寵物 Pet	11	6	0.545	0.000	0.267	5.095 (±0.699)
HKC007	酒精飲料 Alcohol Drink	16	11	0.688	0.042	0.308	4.384 (±1.011)
HKC008	罪行 Crime	17	12	0.706	0.058	0.217	4.829 (±0.740)
HKC009	建築材料 Construction Material	12	10	0.833	0.083	0.267	4.767 (±0.683)
HKC010	布料 Fabric	15	14	0.933	0.092	0.242	4.160 (±0.496)
HKC011	化粧品 Makeup Product	14	7	0.500	0.025	0.267	4.611 (±0.496)
HKC012	鳥類 Bird	25	21	0.800	0.067	0.258	3.808 (±1.120)
HKC013	乳製品 Dairy Product	12	6	0.500	0.042	0.267	5.533 (±0.448)
HKC014	舞蹈 Dance	14	10	0.714	0.033	0.233	3.796 (±0.649)
HKC015	水果 Fruit	18	9	0.500	0.000	0.275	5.292 (±0.553)
HKC016	消防器材 Firefighting Supply	17	9	0.529	0.017	0.242	4.100 (±0.833)
HKC017	花 Flower	24	15	0.625	0.025	0.258	4.160 (±0.790)
HKC018	民間藝術 Folk Art	20	20	1.000	0.150	0.200	4.108 (±0.670)
HKC019	野生動物 Wild Animal	27	19	0.704	0.033	0.242	4.294 (±0.718)
HKC020	疾病 Disease	24	24	1.000	0.100	0.233	4.563 (±1.039)
HKC021	茶葉 Tea	16	10	0.625	0.017	0.200	4.434 (±1.021)
HKC022	家俬 Furniture	18	12	0.789	0.008	0.275	5.625 (±0.601)
HKC023	房屋類型 Housing Type	19	10	0.526	0.017	0.275	4.900 (±0.928)
HKC024	昆蟲 Insect	20	12	0.600	0.017	0.175	4.178 (±1.234)
HKC025	廚房用具 Kitchen Utensil	23	19	0.826	0.067	0.192	5.476 (±0.613)
HKC026	金屬 Metal	16	9	0.529	0.042	0.225	0.245 (±1.101)
HKC027	零食 Snack	19	19	0.947	0.108	0.292	5.042 (±1.003)
HKC028	樂器 Musical Instrument	23	12	0.522	0.000	0.242	3.830 (±0.974)
HKC029	職業 Profession	32	20	0.625	0.000	0.217	5.345 (±0.523)
HKC030	人體器官 Human Organ	25	14	0.560	0.000	0.225	4.938 (±1.036)
HKC031	寶石 Gem	15	9	0.600	0.000	0.000	3.407 (±0.954)
HKC032	祭祀用品 Ancestral Worship Item	14	14	1.000	0.158	0.258	4.943 (±1.363)
HKC033	運動 Sport	28	16	0.571	0.000	0.200	4.704 (±0.702)
HKC034	園藝工具 Gardening Tool	23	19	0.826	0.067	0.167	4.209 (±1.042)
HKC035	玩具 Toy	22	18	0.818	0.067	0.183	4.614 (±0.697)
HKC036	蔬菜 Vegetable	20	13	0.650	0.017	0.167	5.275 (±0.319)
HKC037	武器 Weapon	26	20	0.769	0.050	0.217	4.198 (±1.075)
HKC038	天文現象 Astronomical Phenomena	17	12	0.706	0.058	0.208	4.812 (±0.819)
HKC039	文具 Stationary	18	8	0.444	0.000	0.275	5.378 (±0.709)
HKC040	沐浴用品 Bath Product	13	9	0.692	0.033	0.233	5.346 (±0.661)
HKC041	器皿 Container	13	8	0.615	0.042	0.225	5.388 (±0.595)
HKC042	清潔工具 Cleaning Tool	14	9	0.643	0.008	0.225	5.475 (±0.549)
HKC043	照明工具 Lighting Appliance	14	7	0.500	0.017	0.292	4.800 (±1.095)
HKC044	投資工具 Investment Tool	19	19	1.000	0.175	0.258	4.284 (±0.813)
HKC045	交通工具 Transportation	9	5	0.556	0.008	0.308	5.767 (±0.684)
HKC046	形狀 Shape	14	7	0.500	0.008	0.325	4.850 (±0.295)
HKC047	情緒 Emotional State	28	19	0.679	0.025	0.233	5.752 (±0.506)
HKC048	餐具 Tableware	9	4	0.444	0.000	0.283	5.983 (±0.462)
HKC049	急救用品 First Aid Supply	14	6	0.429	0.025	0.275	4.839 (±0.505)
HKC050	休閒活動 Recreational Activity	31	20	0.645	0.008	0.133	5.385 (±0.788)
HKC051	街頭小食 Street Food	19	8	0.421	0.000	0.317	5.324 (±0.605)
HKC052	語言 Language	12	7	0.583	0.000	0.308	4.650 (±0.897)
HKC053	電影類型 Movie	16	8	0.500	0.000	0.217	5.234 (±0.436)
HKC054	營養補充劑 Nutritional Supplement	25	25	1.000	0.117	0.242	4.212 (±1.089)
HKC055	標點符號 Punctuation Mark	13	7	0.538	0.000	0.325	4.827 (±0.399)
HKC056	貨幣 Currency	14	8	0.571	0.025	0.250	4.461 (±0.553)
HKC057	茶樓點心 Teahouse DimSum	18	9	0.500	0.000	0.300	5.222 (±1.198)
HKC058	燃料 Fuel	13	7	0.538	0.017	0.225	4.515 (±0.650)
HKC059	鞋款 Shoe	12	9	0.750	0.050	0.283	4.763 (±0.932)
HKC060	藝術品 Artwork	26	20	0.654	0.050	0.175	4.675 (±0.740)
HKC061	香港景點 Sightseeing Spot	32	24	0.750	0.033	0.267	4.581 (±0.881)
HKC062	服飾配件 Fashion Accessory	15	13	0.867	0.083	0.150	4.963 (±0.675)
HKC063	年齡組別 Age Group	11	6	0.545	0.000	0.242	5.514 (±0.536)
HKC064	公益團體 NGO	25	18	0.720	0.133	0.042	3.954 (±1.037)
HKC065	電子產品 Electronic Device	12	5	0.417	0.000	0.325	5.833 (±0.641)
HKC066	度量工具 Measuring Tool	15	12	0.800	0.067	0.250	4.330 (±0.899)
HKC067	糖水 Sweet Soup/Tong Sui	18	13	0.647	0.200	0.050	4.750 (±0.998)
HKC068	海洋生物 A Marine Animal	22	13	0.591	0.008	0.133	4.180 (±1.011)
HKC069	時間單位 Time Unit	10	4	0.400	0.025	0.333	5.275 (±1.229)
HKC070	長輩稱呼 Name for Addressing Elder Relatives	26	14	0.538	0.000	0.167	5.146 (±1.062)
HKC071	重量單位 Weight Unit	12	12	1.000	0.100	0.275	4.332 (±1.125)
HKC072	天然能源 Natural Energy Resource	10	7	0.700	0.025	0.192	4.215 (±0.569)

#### Measurement of Category Size and Category Nucleus

Category size is straightforwardly defined as the number of exemplars included in a given category, represented as *Valid Exemplars* in this database. Discrepancies in category size might reflect actual differences in reality (e.g., types of fruit seen and sold in the local markets) or the degree of fine graining of the superordinate-level concept represented by the category label (e.g., “a kind of emotion”) in the lexical inventory.

In our database, a possible alternative measure of category size is the number of exemplars at which the accumulative frequency reaches 0.900, i.e., *Exemplars to 0.90 Coverage* in Table 4. This threshold corresponds to a cut-off rate of 0.100, which excludes highly atypical or idiosyncratic items as “messy residues” and represents a stricter measurement of category size. The ratio (*0.90 Coverage%*) becomes non-negligible with the category statistics mentioned above, although to the best of our knowledge, this has not yet been addressed in the literature. It is possible that a smaller ratio indicates a strong dominance of the top exemplars within the category, a more restricted and unanimous membership, or a smaller category nucleus on the graded structure.

For example, for “a kind of farm animal” (Table 1), just 0.545 of all of the exemplars covered 0.933 of all responses, while the remaining 0.455 exemplars accounted for 0.067 of the members at the other end of category typicality. This means that the first 6 of the 11 exemplars in the category “a kind of farm animal” (cattle, “牛,” *ngau4*; chicken, “雞,” *gai1*; pig, “豬,” *zyu1*; sheep, “羊,” *joeng4*; horse, “馬,” *maa5*; duck, “鴨,” *aap3*) accounted for 93.3% of all of the eligible entries. A person with knowledge of these top exemplars (or highly typical), with half of the category as the category essence or stereotypes, could be considered as being equipped with considerate understanding and word knowledge of the category and its commonly agreed membership.

#### Uneven Knowledge Base

Inevitably, the linguistic realization of the conceptual system in a language community reflects and is shaped by its cultural and social contexts. Conversely, the richness of knowledge about a certain genre may be captured by the abundance of the speakers’ lexical resources of the corresponding categories. In this way, the heterogeneity of categories provides considerate amount of anthropologic semantic details of the language context of the speakers in their everyday lives. *Invalid Exemplars%* in Table 4 can be rendered as a negative indicator of such lexical abundance because most invalid responses are “give-ups” (responses such as “I don’t know” or “−”), repeated instances, or interchangeable rephrases. These invalid responses, which are possibly driven by the no-skipping requirement of the task, reflect a knowledge deficiency for that category or the scarce importance of the genre of knowledge in speakers’ daily communications.

Furthermore, the *Average Familiarity* values and standard deviations in Table 4 provide an overall familiarity estimate for the concepts in the category. The fact that concepts in one category are more consistently familiar across participants than other categories could indicate participants’ higher knowledge of or more frequent exposure to that category, and thus the essentiality of such knowledge.

## Discussion

### Comparisons to Other Norms: Inclusion, Measurements, and Methodologies

Over the years, the Battig and Montague (1969) English norms have been constantly updated and expanded, while researchers have compiled norms in other languages by adapting the category list and using similar methodologies (e.g., Storms, 2001 in Flemish; Marful et al., 2015 in Spanish, and many others). Among them, the norm of Van Overschelde et al. (2004) as an updated English norm, reflected contemporary category membership knowledge and captured the recent cultural changes based on Battig and Montague (1969). It has also been used as a comparable work to many other norm studies (Bueno and Megherbi, 2009 in French). A cross-norm comparison to Van Overschelde et al. (2004) should be representative as the comparison between the current study and the general body of norm studies.

#### Overlapping Categories

Twenty-five categories are common to the two databases, as listed in Table 5. These overlapping categories are used in a wide range of studies and experiments. Contrasting cultural context is apparently a major contributor to the discrepancy on the inclusion of the categories. “A kind of money” in the Van Overschelde et al. (2004) study asked the participants to provide the proper names of United States dollar bills and coins (e.g., dollars, quarters, and dime). There are no such systematically categorical discriminations in HKC. Instead, the HKC study asked the participants to recall their most commonly experienced currencies used in different regions and countries, since international trades and traveling are common experience for the local people. Besides, with almost two decades between the two norming studies, there are inevitably new clusters of concepts emerging, as evidenced by the inclusion of categories such as “HKC064 NGO” and “HKC065 Electronic Devices.”

**TABLE 5:** Comparison with the English norm of Van Overschelde et al. (2004) with list of mutually included categories.

Category in HKC Norming	Category in Van Overschelde et al. (2004)	No. of Exemplars in HKC	No. of Exemplars in Van Overschelde et al. (2004)	Overlapping
HKC002 調味料 Spice	25. A substance for flavoring food	17	25	9
HKC007 酒精飲料 Alcohol Drink	20. An alcoholic beverage	16	19	9
HKC008 罪行 Crime	22. A crime	17	16	8
HKC010 布料 Fabric	9. A type of fabric	15	20	10
HKC012 鳥類 Bird	37. A bird	25	29	13
HKC014 舞蹈 Dance	42. A type of dance	14	22	7
HKC015 水果 Fruit	16. A fruit	18	27	15
HKC017 花 Flower	48. A flower	24	16	6
HKC020 疾病 Disease	49. A disease	24	21	6
HKC022 家俬 Furniture	14. An article of furniture	18	21	9
HKC024 昆蟲 Insect	45. An insect	20	23	15
HKC025 廚房用具 Kitchen Utensil	11. A kitchen utensil	23	19	8
HKC026 金屬 Metal	5. A metal	16	15	9
HKC028 樂器 Musical Instrument	34. A musical instrument	23	25	12
HKC029 職業 Profession	27. An occupation or profession	32	23	14
HKC031 寶石 Gem	1. A precious stone	15	15	8
HKC033 運動 Sport	29. A sport	28	26	8
HKC034 園藝工具 Gardening Tool	69. A gardener’s tool	23	18	8
HKC035 玩具 Toy	41. A toy	22	21	8
HKC036 蔬菜 Vegetable	43. A vegetable	20	25	9
HKC037 武器 Weapon	17. A weapon	26	22	11
HKC045 交通工具 Transportation	39. A transportation vehicle	9	20	6
HKC058 燃料 Fuel	26. A fuel	13	19	9
HKC059 鞋款 Shoe	44. A type of footwear	21	12	6
HKC069 時間單位 Time Unit	2. A unit of time	10	13	9

#### Discrepancy on Measurements and Methodology

The direct measurements given in the norms [*Total* and *Slot 1*, “Total” and “First” in Van Overschelde et al. (2004)] were not defined and computed in an identical way. In Van Overschelde et al. (2004), “Total” was computed “by dividing the number of participants who gave the response by the number of all participants who generated any response” (Van Overschelde et al., 2004, p291), and “First” was computed “by dividing the number of participants who gave the response as the first response by the number of all participants who generated any response” (Van Overschelde et al., 2004, p291–293). More specifically, in a time-limited recall design such as in Van Overschelde et al. (2004)’s English norm, the number of responses from each participant differed; in the current study, all participants generated the same number of responses for a category. Although these two sets of measurements are both indexing the total dominance of an exemplar and the first occurrence of that exemplar, the correlation is not given here since the results and interpretation can be due to the difference in methodology, not in cultural factors.

The position in which an exemplar was recalled was also measured differently. Van Overschelde et al. used “Rank” (i.e., “the mean output position of the response”), whereas the current study reports the probabilities for all the positions (*Slot1*, *Slot2*, and *Slot3*) because there were only three possible positions and participants assigned the positions with intension (driven by the task instruction) of ranking the choices.

This experiment design was adapted from Yoon et al. (2004), a cross language/culture/age norm study which included 105 categories and results from young and old American/Chinese Adults. In HKC norm, three most typical exemplars were provided by 40 participants, in the order of the participants’ subjective ranking of typicality. In Van Overschelde et al. (2004), for each category at least 600 participants gave their responses within the 30s-time limitation, and the norm used a cut-off rate at 0.05 of the participants (i.e., responses mentioned by less than 0.05 participants were discarded from the final database). However, as shown in Table 5, the counts of exemplars generated in the two norms in these mutually included categories are rather comparable, despite the gap between the numbers of participants; there are also considerate proportions of overlapping exemplars, as 0.506 (±0.175, range = [0.250 −0.900]) of the exemplars in HKC categories can also be found in the corresponding categories of Van Overschelde et al. (2004). As for the other non-overlapping half of exemplars, it is tempting to interpret the discrepancy as the cultural/lexical difference affecting the scopes of categories in the two norms (thus two languages); yet it should be noted that it is unclear whether this currently observed discrepancy, or any further comparison results between the current study and other norms using a time-restricted task design, might be also due to the methodological differences.

#### Concepts and Translation

The overlapping exemplars are not identified as one-to-one word pairs using direct translations (Table 6). The different ways of projecting and conceptualizing reality may account for the referring complications: a word in Hong Kong Cantonese may have more than one English translation, and vice versa. For example, for “HKC022 家俬 Furniture” and “14. An article of furniture,” “沙發” (*saa1 faat3*) has two corresponding exemplars, i.e., both “couch” and “sofa” in “14. An article of furniture,” and both “煤油” (*mui4 jau4*) and “火水” (*fo2 seio2*) have “kerosene” as a comparable word in the category of “Fuel,” with “火水” being more colloquial in Hong Kong Cantonese.

**TABLE 6:** Comparison with the English norm of Van Overschelde et al. (2004), showing the overlapping exemplars in the mutually included categories.

Word Code	Exemplar in HKC	in Van Overschelde et al. (2004)
		
**HKC002 調味料 Spice – 25. A substance for flavoring food**		
HKC00201	糖	Sugar(s)
HKC00202	鹽	Salt
HKC00203	胡椒	Pepper
HKC00204	豉油	Soy sauce
HKC00205	辣椒	Paprika; hot sauce
HKC00206	醋	Vinegar
HKC00207	油	Oil(s)
HKC00209	香草	Vanilla
HKC00210	茄醬	Ketchup
**HKC007 酒精飲料 Alcohol Drink – 20. An alcoholic beverage**		
HKC00701	啤酒	Beer
HKC00702	紅酒	Wine
HKC00704	威士忌	Whiskey
HKC00705	伏特加	Vodka
HKC00706	果酒	Wine cooler(s)
HKC00707	雞尾酒	Margarita(s); Martini
HKC00709	烈酒	Liquor(s)
HKC00711	香檳	Champagne
HKC00714	琴酒	Gin
**HKC008 HKC008 罪行 Crime – Crime – 22. A crime**		
HKC00801	偷竊	Stealing/theft/robbery; larceny
HKC00802	謀殺	Murder/killing
HKC00803	強姦	Rape
HKC00804	搶劫	Stealing/theft/robbery
HKC00805	傷人	Battery
HKC00807	詐騙	Arson
HKC00813	綁架	Kidnapping
HKC00815	藏毒	Drug use/possession
**HKC010 布料 Fabric – 9. A type of fabric**		
HKC01001	棉布	Cotton
HKC01002	絲綢	Silk
HKC01003	麻布	Linen
HKC01004	尼龍	Nylon
HKC01005	羊毛	Fleece; wool
HKC01007	絨布	Flannel
HKC01010	牛仔	Denim; jeans
HKC01012	纖維	Rayon
HKC01014	蕾絲	Lace
HKC01015	皮革	Leather
**HKC012 鳥類 Bird – 37. A bird**		
HKC01201	麻雀	Sparrow(s)
HKC01203	烏鴉	Crow(s)
HKC01204	白鴿	Dove; pigeon(s)
HKC01205	鸚鵡	Parrot; parakeet
HKC01207	企鵝	Penguin
HKC01209	雞	Chicken
HKC01211	鵰	Eagle
HKC01212	蜂鳥	Hummingbird
HKC01213	黃鶯	Oriole
HKC01215	貓頭鷹	Owl(s)
HKC01218	海鷗	Seagull(s)
HKC01219	鴕鳥	Ostrich
HKC01225	知更鳥	Mockingbird; robin
**HKC014 舞蹈 Dance – 42. A type of dance**		
HKC01401	芭蕾舞	Ballet
HKC01402	拉丁舞	Tango; salsa; cha cha; mambo
HKC01403	街舞	Hip hop; break
HKC01405	爵士	Jazz
HKC01406	社交舞	Waltz; ballroom; foxtrot
HKC01407	現代舞	Modern
HKC01413	踢踏舞	Tap
**HKC015 水果 Fruit – 16. A fruit**		
HKC01501	蘋果	Apple
HKC01502	香蕉	Banana
HKC01503	西瓜	Watermelon
HKC01504	橙	Orange
HKC01505	士多啤梨	Strawberry
HKC01506	芒果	Mango
HKC01507	提子	Grape
HKC01508	梨子	Pear
HKC01510	檸檬	Lemon
HKC01511	桃	Peach
HKC01512	藍莓	Blueberry
HKC01513	櫻桃	Cherry
HKC01515	木瓜	Papaya
HKC01517	菠蘿	Pineapple
HKC01518	橘子	Tangerine
**HKC017 花 Flower – 48. A flower**		
HKC01701	玫瑰	Rose
HKC01703	百合	Lily
HKC01705	蘭花	Orchid
HKC01709	水仙	Daffodil
HKC01711	向日葵	Sunflower
HKC01712	康乃馨	Carnation
**HKC020 疾病 Disease – 49. A disease**		
HKC02001	感冒	Flu; cold
HKC02002	癌症	Cancer
HKC02003	心臟病	Heart disease
HKC02005	糖尿病	Diabetes
HKC02010	愛滋	AIDS/HIV
HKC02023	天花	Smallpox
**HKC022 家俬 Furniture – 14. An article of furniture**		
HKC02201	沙發	Couch; sofa
HKC02202	椅子	Chair
HKC02204	桌子	Table
HKC02205	床	Bed
HKC02206	衣櫃	Armoire
HKC02207	書桌	Desk
HKC02208	書櫃	Bookshelf
HKC02215	梳妝台(檯)	Dresser
HKC02216	廚櫃	Cabinet
**HKC024 昆蟲 Insect – 45. An insect**		
HKC02401	蝴蝶	Butterfly
HKC02402	螞蟻	Ant
HKC02403	蜜蜂	Bee
HKC02404	甲蟲	Beetle
HKC02405	蟑螂	Roach
HKC02406	蜻蜓	Dragonfly
HKC02407	烏蠅	Fly
HKC02408	蚊（子）	Mosquito
HKC02409	毛蟲	Caterpillar
HKC02411	蜘蛛	Spider
HKC02412	草蜢	Grasshopper
HKC02413	螳螂	Praying mantis
HKC02416	蜈蚣	Centipede
HKC02417	蟋蟀	Cricket
HKC02419	蚤	Flea
**HKC025 廚房用具 Kitchen Utensil – 11. A kitchen utensil**		
HKC02501	菜刀	Knife
HKC02502	鍋子	Pot
HKC02505	砧板	Cutting board
HKC02508	湯匙	Spoon
HKC02512	叉	Fork
HKC02515	碗	Bowl
HKC02518	杯子	Cup
HKC02521	碟	Plate
**HKC026 金屬 – Metal – 5. A metal**		
HKC02601	銅	Copper
HKC02602	金	Gold
HKC02603	鐵	Iron
HKC02604	銀	Silver
HKC02605	鋼	Steel
HKC02607	鉛	Lead
HKC02610	鋅	Zinc
HKC02612	鈦	Titanium
HKC02616	錫	Tin
**HKC028 HKC028 樂器 Musical Instrument – 34. A musical instrument**		
HKC02801	鋼琴	Piano
HKC02802	結他	Guitar
HKC02803	長笛	Flute
HKC02804	小提琴	Violin
HKC02807	口琴	Harmonica
HKC02808	豎琴	Harp
HKC02809	單簧管	Clarinet
HKC02810	大提琴	Cello
HKC02811	色士風	Sax(ophone)
HKC02816	風琴	Organ
HKC02819	小號	Trumpet
HKC02821	大號	Tuba
**HKC029 職業 Profession – 27. An occupation or profession**		
HKC02901	老師	Teacher
HKC02902	醫生	Doctor
HKC02903	警察	Policeman
HKC02904	律師	Lawyer
HKC02905	消防員	Fireman
HKC02906	護士	Nurse
HKC02907	廚師	Cook
HKC02908	球員	Athletes
HKC02909	會計	Accountant
HKC02912	學生	Student
HKC02914	科學家	Scientist
HKC02921	工程師	Engineer
HKC02923	教授	Professor
HKC02924	助理	Secretary
**HKC031 寶石 Gem – 1. A precious stone**		
HKC03101	鑽石	Diamond
HKC03102	紅寶石	Ruby
HKC03103	藍寶石	Sapphire
HKC03104	水晶	Amethyst
HKC03106	綠寶石	Emerald
HKC03108	翡翠	Jade
HKC03112	珍珠	Pearl
HKC03114	石榴石	Garnet
**HKC033 運動 Sport – 29. A sport**		
HKC03301	跑步	Running
HKC03302	足球	Football
HKC03303	籃球	Basketball
HKC03304	游水	Swimming
HKC03307	羽毛球	Badminton
HKC03315	排球	Volleyball
HKC03317	網球	Tennis
HKC03319	壘球	Softball
**HKC034 園藝工具 Gardening Tool – 69. A gardener’s tool**		
HKC03402	泥鏟	Trowel
HKC03405	手套	Glove(s)
HKC03407	泥耙	Rake
HKC03408	泥土	Dirt/soil
HKC03409	水桶	Bucket(s)
HKC03410	鋤	Hoe
HKC03411	割草機	Lawnmower
HKC03414	水管	Water hose
**HKC035 玩具 Toy – 41. A toy**		
HKC03501	公仔	Stuffed animals
HKC03502	玩具車	Cars
HKC03504	搖搖	Yo-yo
HKC03505	洋娃娃	Dolls; Barbie dolls
HKC03506	積木	Blocks
HKC03509	皮球	Balls
HKC03510	拼圖	Puzzles
HKC03511	電腦	Computer
**HKC036 蔬菜 Vegetable – 43. A vegetable**		
HKC03601	白菜	Cabbage
HKC03603	生菜	Lettuce
HKC03604	蕃茄	Tomato, tomatoes (20)
HKC03605	蘿蔔	Radish
HKC03606	西芹	Celery
HKC03607	西蘭花	Broccoli
HKC03610	椰菜	Cauliflower
HKC03611	青瓜	Cucumber
HKC03614	菠菜	Spinach
**HKC037 武器 Weapon – 17. A weapon**		
HKC03701	刀	Knife
HKC03702	手槍	Gun
HKC03703	劍	Sword
HKC03704	斧頭	Axe
HKC03705	炸彈	Bomb
HKC03706	弓	Bow
HKC03709	拳頭	Fist
HKC03710	手榴彈	Grenade
HKC03715	矛	Spear
HKC03717	雙節棍	Nunchucks
HKC03724	棍	Stick
**HKC045 交通工具 Transportation – 39. A transportation vehicle**		
HKC04501	巴士	Bus
HKC04502	地鐵	Subway
HKC04503	的士	Taxi/cab
HKC04506	火車	Train(s)
HKC04508	私家車	Car(s)
HKC04509	飛機	(Air)plane
**HKC058 燃料 Fuel – 26. A fuel**		
HKC05801	煤	Coal
HKC05802	石油	Oil
HKC05803	天然氣	Natural (gas)
HKC05804	汽油	Gasoline
HKC05805	木柴	Wood
HKC05806	柴油	Diesel
HKC05808	化石	Fossil
HKC05809	火水	Kerosene
HKC05812	煤油	Kerosene
**HKC059 鞋款 Shoe – 44. A type of footwear**		
HKC05901	波鞋	Sneaker; tennis; Nikes; Adidas
HKC05903	拖鞋	Slipper; flip flops
HKC05904	高跟鞋	High heels; pumps
HKC05905	跑鞋	Running shoes
HKC05906	涼鞋	Sandal
HKC05908	靴子	Boot
**HKC069 時間單位 Time Unit – 2. A unit of time**		
HKC06901	小時	Hour
HKC06902	分鐘	Minute
HKC06903	秒鐘	Second
HKC06904	年	Year
HKC06905	月	Month
HKC06906	毫秒	Millisecond
HKC06907	日	Day
HKC06908	世紀	Century
HKC06910	星期	Week

In all, on the category level, the HKC norm covered a considerable range of categories that were in common with the English norm of Van Overschelde et al. (2004) and the cross-culture norm of Yoon et al. (2004); on the exemplar level, overlapping exemplars are identified with the referring discrepancy of the concept observed.

### Potential and Benchmarks

In addition to the current representation of the categories, the exemplars, and the descriptive statistics, the database could provide primary training data for a more complex model with additional variables explored. The data presented in the current study is rather straight forward, as categories independent of each other and the exemplars are associated by their mutual categorical information. To further examine an interconnected semantic knowledge structure, more variables such as semantic relatedness of the exemplars and categorical feature analysis would be necessary, such that both intra-category exemplar relations and inter-category relations would be captured. This approach would provide a more sophisticated analysis of the semantic network of Hong Kong Cantonese, with an exploration of the concept clustering and interconnections between categories and concepts.

The processing efficiency of the highly typical exemplars suggests that categorical typicality imposes a spontaneous contextual prime on an exemplar, which can be considered as the stored semantic information about an exemplar. If we accept the hypothesis that *Slot1* measures a kind of instant typicality, then this time-sensitive quality may be exploited in psychophysiological experiments to investigate the online processing of exemplars with congruent and incongruent categorial information primes. This type of investigation could be achieved by monitoring brain activity using technologies such as electroencephalography and event related potentials (Stuss et al., 1988; Kounios and Holcomb, 1992; Kutas and Iragui, 1998; Federmeier and Kutas, 1999). Furthermore, the data collected from young, healthy adults can serve as a benchmark for studies of other age groups, namely older adults and children, and of patients with cognitive deficits. As the semantic knowledge is generally preserved in the elder population (e.g., Park et al., 2002), this database provides resources in examining neural mechanisms of word retrieval for Cantonese-speaking elderlies. On the other hand, comparisons between the responses provided by neurologically impaired subjects and the database may reveal the domain-specific degeneration of semantic knowledge. The database may also benefit developmental studies examining how children establish lexical inventories by observing category and exemplar learning.

## Conclusion

This paper presents a norming study of category instance production for 72 natural semantic categories in modern Hong Kong Cantonese, with instance probability and familiarity rating results. Total exemplar production probability and the probabilities of different positions of occurrence provide a detailed statistical description of instance typicality. In addition, word familiarity is provided for each included exemplar as independent words from their categorical information. The split-half correlation as the reliability measurements confirms that the norming results are reliable and consistent. The database addresses the lack of a Hong Kong Cantonese category norming database and opens up research potential in multiple fields.

## Data Availability Statement

The original contributions presented in the study are included in the article/supplementary material, further inquiries can be directed to the corresponding author/s.

## Ethics Statement

The studies involving human participants were reviewed and approved by Ethics Committee, City University of Hong Kong. The patients/participants provided their written informed consent to participate in this study.

## Author Contributions

BL contributed to data collection, formal analysis, and writing—original draft. QL contributed to data collection and formal analysis. HYM contributed to formal analysis and visualization. OT contributed to conceptualization, funding acquisition, and resources. C-MH contributed to conceptualization, investigation, and methodology. H-WH contributed to conceptualization, formal analysis, investigation, methodology, project administration, supervision, validation, writing—original draft, and writing-review and editing. All authors contributed to the article and approved the submitted version.

## Conflict of Interest

The authors declare that the research was conducted in the absence of any commercial or financial relationships that could be construed as a potential conflict of interest.

## Publisher’s Note

All claims expressed in this article are solely those of the authors and do not necessarily represent those of their affiliated organizations, or those of the publisher, the editors and the reviewers. Any product that may be evaluated in this article, or claim that may be made by its manufacturer, is not guaranteed or endorsed by the publisher.

## Appendix

**Appendix 1:** 12 categories that were excluded from the final list because the invalid responses exceeded 50% of the total responses collected in the categories.

Category Name in HKC	Category Name in English
魚類	A Kind of Fish
電視節目	A TV program
中藥材	A Herb in Chinese Medicine
天然地形	A Natural Geographical Feature
刊物類別	A Type of Publication
本地地名	A Name of Local Place
參考書	A kind of Reference Book
音樂類型	A Kind of Music
學系	A Department in University
藝術品	A Piece of Artwork
五金用品	A Piece of Hardware Supplies
康樂項目	A Recreational Activity
